# Diet, Nutrition, and Oral Health in Older Adults: A Review of the Literature

**DOI:** 10.3390/dj11090222

**Published:** 2023-09-19

**Authors:** Alice Kit Ying Chan, Yiu Cheung Tsang, Chloe Meng Jiang, Katherine Chiu Man Leung, Edward Chin Man Lo, Chun Hung Chu

**Affiliations:** Faculty of Dentistry, The University of Hong Kong, Hong Kong 999077, China; dralice@hku.hk (A.K.Y.C.); elvist@hku.hk (Y.C.T.); cmjiang@hku.hk (C.M.J.); kcmleung@hku.hk (K.C.M.L.); hrdplcm@hku.hk (E.C.M.L.)

**Keywords:** older adult, elderly, oral health, prevention, caries, periodontal disease, diet

## Abstract

Diet, nutrition, and oral health are closely linked. Malnutrition is a challenging health concern in older adults that is associated with physical decline affecting their daily activities and quality of life. The aim of this review is to provide an evidence-based summary of the relationship between diet and nutrition and oral health in older adults and its implications. The World Health Organization has declared healthy ageing a priority of its work on ageing. The American Dental Association confirmed the bidirectional relationship between diet and nutrition and oral health. The literature shows that diet and nutrition are related to oral diseases, including dental caries, periodontal diseases, tooth wear, and even oral cancer. Insufficient nutritional intake and poor dietary habits increase the risk of oral diseases, such as dental caries, in older adults. On the other hand, in older adults, poor oral conditions such as periodontal disease may induce pain, infection, and tooth loss, affecting nutritional intake. Surveys have shown that older adults, in particular, those in disadvantaged communities, suffered from nutritional deficiencies or imbalances affecting their oral health. In addition, the current literature shows that malnutrition is associated with frailty, hospitalization, mortality, and morbidity. Good oral health and functional dentition are essential to maintain sufficient nutritional intake among older adults and reduce the risk of malnutrition. Therefore, integrating oral health into general health care service in older adults is imperative to improve their nutritional and oral health status to achieve healthy ageing.

## 1. Introduction

The World Health Organization listed nutritional health and oral health status as pivotal concerns in older adults and the key elements in achieving healthy ageing [1,2]. The World Report on Ageing and Health 2015 by the World Health Organization emphasized that healthy ageing is not only being disease-free but also maintaining the functional ability for older adults to be and do what they value [3]. FDI World Dental Federation defined oral health as “multifaceted and included the ability to speak, smile, smell, taste, touch, chew, swallow, and covey a range of emotions through facial expressions with confidence and without pain, discomfort, and disease” [4]. This new definition of oral health acknowledged the importance of oral health in nutritional intake. A healthy lifestyle with adequate nutrition intake can prevent or delay the progression of a number of chronic diseases [3]. Therefore, good nutrition and oral health are essential for healthy ageing in older adults [3].

The risk of having poor nutritional and oral health status increases with age due to physiological age changes, chronic medical conditions, and medications [1,5,6]. Nutritional intake and oral health share some common social determinants of health in older adults [1,7]. Older adults exhibit a decline in their cognitive and functional abilities as they age [8]. They may gradually lose the self-care abilities of preparing food, cooking, and brushing their teeth [1,9]. Physiological age changes lead to taste buds and salivary gland atrophy in older adults [9,10]. Changes in taste buds may alter their taste sensation, leading to changes in food preference and decreased appetite [11]. Salivation facilitates chewing and swallowing, and hyposalivation may make chewing and swallowing difficult, leading older adults to have a diet with softer food and less fiber [11]. Hyposalivation also increases the risk of developing oral diseases such as dental caries and periodontal disease [11]. Chronic medical conditions in older adults, such as arthritis and Parkinson’s disease, further reduce their self-care abilities as well as their nutrition and oral health care [9]. They may need assistance in buying food or attending dental services.

The medications associated with these medical conditions not only affect nutritional absorption in the digestive system but also increase the risk of hyposalivation [11]. Social determinants of health, such as living alone, low income, and low health literacy, make food supply and dental health care less accessible, affordable, and available to older adults [1,7]. Therefore, older adults have an increased risk of poor nutritional and oral health than other age groups.

The American Dental Association confirmed the bidirectional relationship between diet and nutrition and oral health [12]. Nutritional intake and dietary habits affect the risk of oral diseases, including dental caries, periodontal diseases, tooth wear, and oral cancer. In older adults, poor oral conditions, such as dental caries, periodontal disease, worn dentition, dry mouth, and ill-fitted dentures, may induce pain, infection, and tooth loss, affecting nutritional intake [13,14]. Older adults are at risk of nutritional deficiencies or imbalances, which affect their oral and systemic health [14,15] and may lead to an unhealthy loss of muscle, resulting in a decline in functional status (ability to move and chew), independence, and immune function [15].

The integration of oral health into general health care services is paramount to ensure good nutrition and oral health status in older adults [16]. A multidisciplinary team should conduct a comprehensive geriatric assessment and formulate a coordinated, integrated, and patient-based treatment plan for all older adults to maintain their oral and general health until the end of their lifetime [16]. The aim of this narrative review is to provide an evidence-based summary of the association between diet and nutrition and oral health in older adults and the implications for improving their nutritional and oral health status. This review is based on English-language publications, including clinical studies and reviews, identified in the PubMed and Google Scholar databases, and the information available up to May/2023 from the websites of international organizations such as the World Health Organization, FDI World Dental Federation, and American Dental Association on diet and nutrition and oral health in older adults. The search was conducted using the keywords “diet”, “nutrition”, “nutrient”, “nutritional intake”, “malnutrition”, “oral health”, and “oral disease”.

## 2. The Impact of Diet and Nutrition on Oral Health

“Diet” refers to the food and drink an individual consumes, as well as the mental and physical circumstances (frequency and methods of intake) related to eating. “Nutrition” refers to macronutrients (protein, carbohydrates, and fat) and micronutrients (vitamins and minerals) that the body needs [12]. The intake of certain types of macronutrients or micronutrients as well as dietary behavior, such as the frequency of intake, may impact oral health [17]. Malnutrition, which the World Health Organization defined as “deficiencies or excesses in nutrient intake, imbalance of essential nutrients or impair nutrient utilization”, is also associated with oral health in older adults [18].

### 2.1. Dental Caries

Dental caries results from the demineralization of dental hard tissues due to the acidic by-products the bacteria in the biofilm (dental plaque) produces via fermentation of dietary carbohydrates [19]. Half of the global older adult population has untreated dental caries, and diet is one of the caries risk factors in older adults [20]. There is unequivocal evidence showing that fermentable carbohydrates (sugars and starch) are essential in caries initiation and progression [17]. Caries risk varies with the type and amount of carbohydrates and the intake frequency [17].

The World Health Organization recognized that free sugars are the key elements in caries development, and it defined free sugars as “all monosaccharides and disaccharides added to foods and drinks by the manufacturer, cook or consumer, and sugars naturally present in honey, syrups, fruit juices and fruit juice concentrates” [21]. Among all free sugars, sucrose should receive more attention, because it can be rapidly converted into acid, resulting in a profound drop in pH [17,22]. Moreover, sucrose can be synthesized into extracellular glucans, fructans, and intracellular storage compounds promoting the formation of biofilm with lower concentrations of the buffering elements such as calcium, phosphorus, and fluoride [17,22]. There is moderate evidence showing that a diet with free-sugar intake of more than 10% of total energy intake increases the risk of dental caries [17,23]. Therefore, the World Health Organization recommends free-sugar intake below 10% of total energy intake and to reduce free-sugar intake throughout the life course [24]. Older adults experience a decline in taste perception of salty, sweet, and umami with age and tend to choose stronger flavours, with a greater consumption of sweet and salty foods [25], which may increase sugar intake. A study showed that intake of sucrose in coffee or tea was associated with the increment of root surface caries in community-dwelling older Japanese adults [26].

Frequency of sugar intake is another crucial factor in caries development [27]. A drop in pH following sugar intake can last more than 30 min [27]. Therefore, if sugar intake is frequent, the pH of dental biofilm will remain low constantly, increasing the risk of caries development [27]. Older adults tend to have smaller portions of food during meals due to reduced appetite resulting from age changes, systemic diseases, and medications, but they may form snacking habits between meals [11,28]. Studies have found that over 84% of American older adults have snacking habits; studies have also revealed that the snacking habit helps these older adults acquire an adequate daily nutritional intake [29]. However, these studies did not consider the possible side effects of frequent snacking habits on oral health.

### 2.2. Periodontal Disease

Periodontal disease is the inflammation of the periodontium, in which dental plaque elicits a series of host responses to mediate inflammation, resulting in tissue destruction characterized as pocket formation, gingival recession, and alveolar bone resorption in susceptible individuals [30,31]. Periodontal disease is cumulative and prevalent in old age, affecting more than 60% of the global older adult population [32]. There is emerging evidence indicating periodontal disease is associated with diet [17]. Certain dietary patterns and nutrient intake can trigger or regulate immune-mediated inflammatory responses, influencing the development of periodontal disease [33]. A diet rich in carbohydrates and saturated fats is proinflammatory and may increase the risk of periodontal disease, whereas one rich in polyunsaturated fatty acids, fiber, fruits, vegetables, antioxidant micronutrients, and calcium is anti-inflammatory and may decrease the risk of periodontal disease [17,33].

A systematic review found that the risk of periodontal disease was inversely associated with the intake of fatty acids, vitamin C, vitamin E, beta-carotene, fiber, dairy calcium, fruits, and vegetables in community-dwelling older adults [33]. The higher intake of n-3 polyunsaturated fatty acids and dietary antioxidants such as vitamin C, vitamin E, and beta-carotene have been shown to retard the progression of periodontal disease, with reduced number of teeth having clinical attachment loss in Japanese older adults [34,35]. N-3 polyunsaturated fatty acids increase pro-resolving lipid mediators and thereby regulate the destructive inflammatory response, whereas dietary antioxidants mitigate the level of oxidative stress and hence reduce inflammation in periodontal tissues [34,35]. Increased intake in total dairy calcium from dairy products, especially from milk and fermented foods, reduced the risk of periodontitis in Danish older adults [36]. However, most studies were assessed as weak in quality because they were based on small sample sizes [33]. Recent studies have focused on the use of adjunctive ingestion of fruit and vegetable extracts and probiotics to improve the clinical outcomes following periodontal therapy, yet the evidence remains weak [17].

### 2.3. Tooth Wear

The prevalence of tooth wear increases with age: up to 17% at the age of 70 [9]. Tooth wear includes attrition, abrasion, erosion, or any combination thereof [9]. Dental erosion is strongly linked to diet [9,37]. The American Dental Association defined dental erosion as the progressive and irreversible loss of dental hard tissue caused by a chemical process of acid dissolution that does not involve bacteria [37]. The source of acid can be intrinsic, often due to gastric reflux, or extrinsic, due to the consumption of acidic food/beverage, such as carbonated/soft drinks and acidic fruit juice [37]. The pH of food/beverage affects their erosive potential on dentition [38]. Teeth erode in the pH of 2.0 to 4.0, and food/beverage is considered to be erosive to dentition if its pH is lower than 4.0 [38]. A study assessed the pH of 379 commercially available beverages in the United States and detected the lowest pH of 2.4 in lemon juice [38].

A positive relationship between 100% fruit juice consumption and dental erosion has been reported in the literature; studies have found that frequent consumption of natural fruit juice increased the risk of dental erosion at an odds ratio of 1.2 [39,40]. Fruits play an important role in a healthy dietary pattern, as proposed in the Dietary Guidelines for Americans, and in achieving healthy ageing in older adults [41]. Consumption of various acidic fruit juices (pomegranate, cherry, and beetroot) has been found to improve numerous health conditions, including cognitive function, low-density lipoprotein cholesterol level, and blood pressure in older adults [42,43]. Many older adults were encouraged to increase their fruit or fruit juice consumption as a healthy lifestyle yet were not informed about the deleterious effect of fruit or fruit juice on their dentition [44].

A study found that timing of fruit intake was crucial in tooth wear progression, with a significantly increase in the odds ratio (3.64) for fruit intake between meals when compared to fruit intake with meals [45]. Older adults can be advised about the proper dietary fruit intake habits to avoid tooth wear progression. Dietary supplements may be prescribed to older adults who are at risk of nutritional deficiency. Of all the supplements, chewable vitamin C (ascorbic acid) tablets increased the risk of dental erosion at an odds ratio of 1.16 [46]. However, only limited studies were carried out in older adults to investigate the association between diet and nutrition and tooth wear.

### 2.4. Oral Cancer

Oral cancer ranks as the 13th most common cancer worldwide, and alcohol consumption is one of the leading causes of oral cancer [47]. The risk of oral cancer increases with age and is therefore higher in adults aged 65 and above [9,48]. There is robust evidence showing a significant increased risk of oral cancer with increased alcohol consumption [48]. Alcohol consumption was associated with head and neck cancer at an odds ratio of 2, if three or more drinks per day were consumed, when compared to no alcohol consumption [49]. It was believed that acetaldehyde, the major and most toxic metabolite of alcohol, interfered with DNA synthesis and repair and triggered a carcinogenic cascade [48]. Although evidence of the link between diet and nutrition and oral cancer was limited, findings from studies have, generally, consistently indicated that greater consumption of non-starchy vegetables decreased the risk of oral cancer [48]. The proposed mechanism was the antitumorigenic effects of the wide range of nutrients and phytochemicals such as carotenoids, vitamin A, vitamin C, vitamin E, and flavonoids contained in vegetables, yet further studies are needed to explore their association and underlying mechanisms [48].

**Figure 1:**
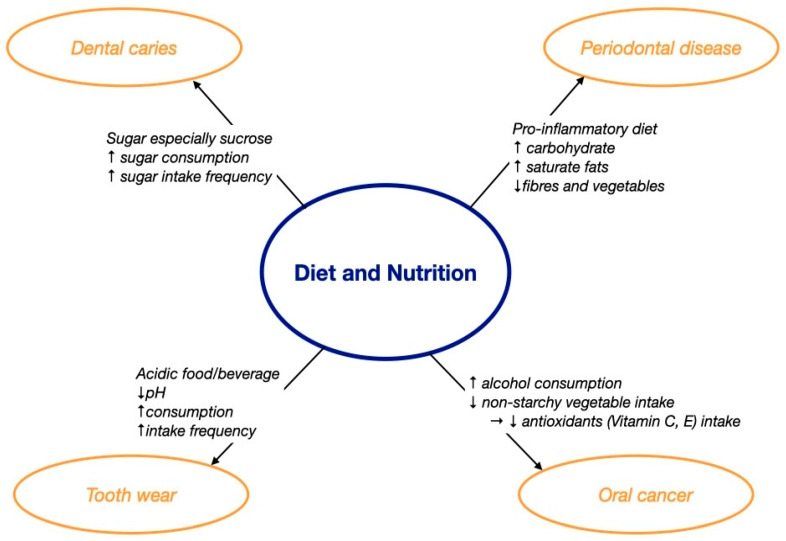
The effect of diet and nutrition on oral health. ↑ increase; ↓ decrease; → lead to.

The literature has shown that oral diseases, including dental caries, periodontal disease, tooth wear, and oral cancer, are related to diet and nutrition [14]. Figure 1 summarizes how diet and nutrition affected oral health, and Table 1 lists the clinical studies and systematic reviews about the effect of diet and nutrition on oral health. Although there were numerous clinical studies on the effect of diet on periodontal disease, the evidence was from cross-sectional or longitudinal studies only. Moreover, the clinical studies investigating the effect of diet on tooth wear and oral cancer were mainly conducted on adults rather than focusing on older adults. It is essential to conduct more high-level clinical studies to confirm the casual relationship between diet and oral disease in older adults.

## 3. The Impact of Oral Health on Diet and Nutrition

The oral cavity is at the first part of the digestive tract and is responsible for chewing, salivation, and swallowing to transport food bolus to the stomach for nutritional intake [50]. In older adults, several common dental problems such as dental caries, periodontal disease, tooth wear, and oral cancer may induce pain, infection, and tooth loss and jeopardize the normal digestive process for nutritional intake [9]. Salivation is important for taste sensation, bolus formation, and swallowing. Hyposalivation, a common oral condition in older adults, may hence affect nutritional intake [9]. If the reduced nutritional intake is left uncontrolled, it may lead to nutritional deficiency, which is associated with frailty, mortality, and morbidity in older adults [13].

### 3.1. Pain

Dental caries, periodontal disease, and tooth wear may induce dentine hypersensitivity or even pain, which limits food choice, reduces chewing efficiency, and hinders nutritional intake [13]. Home care older adults in Finland who presented with toothache had an increased risk of oral health-related chewing problems (odds ratio of 10.3) [51]. Another study conducted in a nursing home located at Germany found that older adults with discomfort upon chewing demonstrated more food avoidance [52]. A recent study showed that pain upon chewing due to ill-fitting dentures, dental caries, or dentine hypersensitivity was the determinant of the incidence of nutritional deficiency in community-dwelling older adults at a hazard ratio of 2 and suggested that diagnosis and management of the underlying oral conditions may aid the prevention of nutritional deficiency in older adults [13].

### 3.2. Infection

Poor oral health can affect general health [9]. Oral infection, such as periapical inflammation and periodontal disease, may induce chronic low-grade inflammation systemically, leading to anorexia with reduced food intake and metabolic alterations with increased muscle catabolism [53]. Chronic low-grade inflammation increases the circulating level of interleukin 1, interleukin 6, and tumor necrosis factor alpha which delays the gastric emptying and clamps the small intestinal motility, leading to anorexia, defined as the presence of decreased food intake or poor appetite [54]. Anorexia, a multifactorial condition, affects one fifth of the older adult population and is associated with nutritional deficiency, frailty, morbidity, and mortality in older adults [27]. Systemic inflammation alters metabolism with an increase in muscle catabolism, resulting in a decrease in muscle mass and strength [53]. This impairs the functional status of muscles, reducing the masticatory efficiency and mobility of frail older adults or those with sarcopenia, a pathological condition with progressive and accelerated loss of muscle mass and strength, leading to poor function [55]. There is increasing evidence of the associations among oral health, malnutrition, and sarcopenia in frail older adults, indicating that a multidisciplinary intervention is needed for better care in older adults [55].

### 3.3. Tooth Loss

Teeth are responsible for incising, tearing, and grinding food particles into smaller pieces, which are mixed in the saliva to form a bolus for swallowing [56]. The World Health Organization states that the retention of a minimum number of 20 natural teeth without any prostheses should be maintained to ensure adequate function, and the literature further supports the conclusion that a minimum pair of occluding natural teeth is necessary to provide functional dentition [57,58]. Dental caries and periodontal disease, if left untreated, may eventually lead to tooth loss [9]. The prevalence of tooth loss increases with age, with a peak in incidence at the age of 65 [59]. In 2015–2018, the prevalence of complete tooth loss was 13% in older adults aged 65 or above in the United States, and the situation is likely worse in underdeveloped countries [60,61]. The loss of natural teeth makes mastication more challenging for older adults. Older adults with a lack of functional dentition had an increased risk of chewing disability (odds ratio of 4.7) [62]. Tooth loss also affects swallowing. The bolus size increases as the number of teeth decreases, and the difficulty in swallowing increases as the bolus size increases [63]. Older adults with severe tooth loss tend to avoid harder food such as meats, fruits, and vegetables, which are the main sources of proteins, fiber, minerals, and vitamins [64]. Severe tooth loss resulting in a lack of functional dentition or edentulism affects nutritional intake and increases the risk of nutritional deficiency in older adults by 21% [64].

### 3.4. Hyposalivation

**Figure 2:**
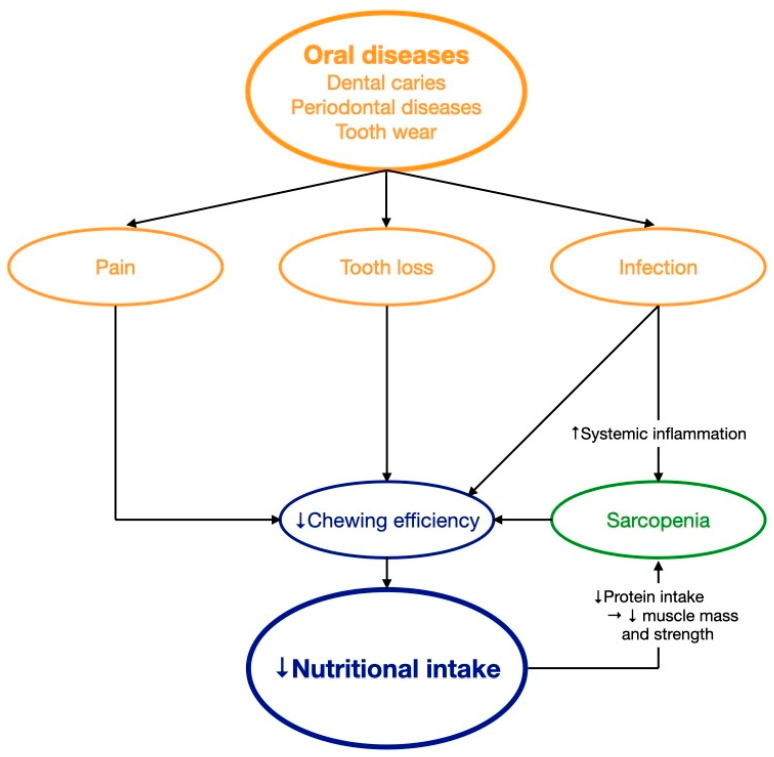
The effect of oral health on nutritional intake. ↑ increase; ↓ decrease; → lead to.

Hyposalivation is a common condition affecting one third of the older adult population [65]. Age changes, chronic medical conditions, and medications contribute to hyposalivation in older adults [9]. Hyposalivation may alter taste sensation and impair swallowing, leading many older adults to choose softer food [11]. A cross-sectional study found that older Japanese adults with hyposalivation had significantly reduced vegetable and fish consumption, leading to lower intake of n-3 polyunsaturated fatty acids, vitamin E, vitamin B6, and folate than those without hyposalivation [66]. Hyposalivation may reduce the retention of removable dentures and, hence, the comfort of denture wearing in older adults during chewing [13]. Pain upon chewing with ill-fitting dentures was a risk factor of nutritional deficiency in older adults [13]. However, the evidence of the association between hyposalivation and malnutrition remains scarce [13]. In general, poor oral health with pain, infection, or tooth loss may reduce nutritional intake and, if uncontrolled, lead to nutritional deficiency with inadequate intake of macronutrients, minerals, and vitamins in older adults. Macronutrients (proteins, carbohydrates, and fiber) are essential in maintaining muscle mass and strength whereas micronutrients (minerals and vitamins) are key elements in regulating immune function [67]. Insufficient intake of macronutrients may lead to unhealthy loss of muscles and increase the risk of developing sarcopenia and frailty [67]. Micronutrient deficiency may impair the immune function, increase the risk of infection, and delay the recovery from disease in older adults [67]. Figure 2 describes how oral health affects nutritional intake.

Table 2 summarizes the literature on the adverse effects of oral conditions on nutritional intake. There is emerging evidence on the effect(s) of both tooth loss and pain on chewing and nutritional intake, yet much came from cross-sectional and longitudinal studies. Moreover, evidence on the effect of hyposalivation on nutritional intake is still scarce.

## 4. Implications for Improving Nutritional and Oral Health in Older Adults

Diet and nutrition and oral health are interrelated. Older adults are at a risk of nutritional deficiency and poor oral health. The World Health Organization and FDI World Dental Federation agreed that integration of oral health care into general health care services for older adults is an imperative strategy to improve their nutritional and oral health status [16]. A multidisciplinary team including oral and health care professionals should collaborate to conduct a comprehensive geriatric assessment comprising the oral, nutritional, and medical status of older adults to formulate a patient-oriented, coordinated, and integrated treatment plan to improve their oral and overall health [69]. Policy makers, international professional organizations, public health professionals, academics, and individual health care professionals can all help advocate this integration [16].

Policy makers should advocate the integration of oral health care into general health care services in older adults with the provision of resources, financial funding, and multidisciplinary clinical settings [16]. Because most health care professionals are unaware of the link between diet and nutrition and oral health, it is essential for international professional organizations to provide education or workshops to raise their awareness and enhance their knowledge and skills on this topic [70]. Public health professionals should conduct community services for the public to educate them about the oral health benefits of maintaining a healthy diet, the side effects of malnutrition on oral and systemic health, and how to obtain healthy and adequate nutrition starting from assessing the nutrition facts label on food packages and the importance of healthy dietary habits.

Academics should provide more evidence to support such integration. Most findings on the association between diet and nutrition and oral health come from cross-sectional studies with small sample sizes. Studies were mainly conducted in a few countries, such as Japan, the United States, and a few European countries. More longitudinal studies with larger sample sizes from different countries should be conducted for a better understanding of the association between diet and nutrition and oral health in older adults [13]. Clinical trials with intervention arms should also be conducted to investigate the effectiveness of various nutritional interventions in improving oral health outcomes in older adults to provide more evidence regarding their clinical uses.

**Figure 3:**
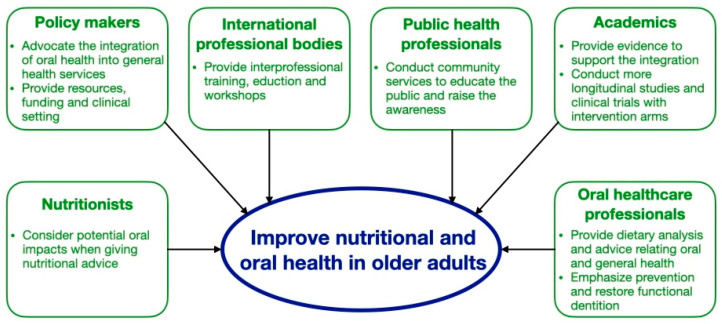
Role of different stakeholders in improving nutritional and oral health in older adults.

Nutritionists should consider the potential impacts on oral health when providing older adults with nutritional advice. Snacking frequency and the cariogenic qualities and acidity of food or drink should all be considered. Milk and dairy products, tea, and high-fiber foods are suggested to be cariostatic and could be recommended as snacking substitutes or alternatives to reduce sugar intake in meals when needed [71]. Older adults should increase the intake of polyunsaturated fatty acids, which are found in high quantities in salmon, nuts, fibers, and vegetables. Further, older adults should consume fruit or fruit juice with meals, rather than separately, to reduce the incidence of dental erosion. Oral health care professionals should conduct dietary analysis in all older adults on a routine basis and provide dietary advice to improve their oral health. They should also be aware of the current evidence-based nutritional recommendations and arrange proper referral to a nutritionist if they notice the older adult may have a risk of nutritional deficiency or imbalance. Figure 3 outlines the role of different stakeholders in improving nutritional and oral health in older adults.

Prevention is better than a cure, so preventive measures should be delivered and reinforced to maintain good oral health and functional dentition in older adults over their life course [7]. Fluoride is well proven for caries prevention, and the use of fluoridated toothpaste twice daily is a simple, convenient, and low-cost caries prevention method for older adults [72]. Prosthodontic rehabilitation can be considered for restoring functional dentition in older adults with severe tooth loss to improve their nutritional status [68]. There is increasing evidence suggesting that prosthodontic treatment in combination with personalized dietary counselling may further improve individuals’ nutritional status [73].

## 5. Conclusions

Diet and nutrition and oral health are interrelated. Proper nutrition and healthy eating habits enhance oral health, and functional and healthy dentition aids in nutritional intake. Nutritional education is paramount to increase the awareness of the public as well as oral and health care professionals. The integration of oral health into general health care services can ensure that all older adults receive coordinated and integrated treatment to maintain oral and general health in their lifetime.

## Figures and Tables

**Table 1:** Systematic reviews and clinical studies of diet and nutrition on oral health (#: study was specified for older adults).

Study Type (Authors, Year)	Title	Main Findings
Dental caries		
Systematic review (Chan et al., 2021) [20]	“A systematic review on caries status of older adults”	Diet was a risk factor for caries in older adults.
Cross-sectional study (Yoshihara et al., 2021) [26]	“Diet and root surface caries in a cohort of older Japanese”	High intake of sucrose in coffee or tea and low milk intake were associated with the increment of root surface caries.
Periodontal diseases		
Systematic review (O’Connor et al., 2020) [33]	“Poor dietary intake of nutrients and food groups are associated with increased risk of periodontal disease among community-dwelling older adults: a systematic literature review”	High intakes of fatty acids, vitamin C, vitamin E, beta-carotene, fiber, calcium, dairy, fruits, and vegetables were inversely associated with the risk of periodontal disease.
Longitudinal study (Iwasaki et al., 2011) [34]	“Dietary ratio of n-6 to n-3 polyunsaturated fatty acids and periodontal disease in community- based older Japanese: a 3-year follow-up study”	High dietary n-6 to n-3 polyunsaturated fatty acids ratio was associated with greater number of periodontal disease events.
Longitudinal study (Iwasaki et al., 2013) [35]	“Dietary antioxidants and periodontal disease in community-based older Japanese: a 2-year follow-up study”	A high intake of antioxidants (vitamin C, vitamin E, α-carotene, and β-carotene) was inversely associated with periodontal disease progression.
Cross-sectional study (Adegboye et al., 2012) [36]	“Intake of dairy products in relation to periodontitis in older Danish adults”	Total dairy foods, milk, and fermented food intakes were associated with reduced risk of periodontitis.
Tooth wear		
Systematic review # (Liska et al., 2019) [39]	“100% fruit juice and dental health: a systematic review of the literature”	Intake of 100% fruit juice could contribute to tooth erosion in adults.
Case-control study # (O’Toole et al., 2017) [45]	“Timing of dietary acid intake and erosive tooth wear: A case-control study”	Acid consumption between meals increased risk of erosive tooth wear in adults (odds ratio: 11.84).
Systematic review # (Li et al., 2012) [46]	“Dietary factors associated with dental erosion: a meta-analysis”	Intake of soft drinks (odds ratio: 2.41) and vitamin C (odds ratio: 1.16) was associated with dental erosion in adults.
Oral cancer		
Systematic review # (Hashibe et al., 2007) [49]	“Alcohol drinking in never users of tobacco, cigarette smoking in never drinkers, and the risk of head and neck cancer: pooled analysis in the International Head and Neck Cancer Epidemiology Consortium”	Alcohol consumption of more than two drinks daily increased risk of head and neck cancer (odds ratio: 2)

**Table 2:** Clinical studies and systematic reviews on the adverse effects of oral conditions on nutritional intake.

Study Type (Authors, Year)	Title	Main Findings
Poor oral health		
Systematic review (Algra et al., 2021) [18]	“The association between malnutrition and oral health in older people: A systematic review”	In older adults, malnutrition was associated with self-perceived poor oral health, hard tissue problems such as few functional teeth, and soft tissue problems such as cracked lips.
Pain on chewing		
Longitudinal study (Kiesswetter et al., 2019) [13]	“Oral health determinants of incident malnutrition in community-dwelling older adults”	Toothache while chewing increased risk of malnutrition of older adults (odds ratio: 2.14).
Cross-sectional study (Salmi et al., 2022) [51]	“Eating problems among old home care clients”	Edentulous older adults (odds ratio: 3.5) and older adults with toothache or denture problems (odds ratio: 10.3) had a higher risk for oral health-related eating problems.
Cross-sectional study (Altenhoevel et al., 2012) [52]	“The impact of self-perceived masticatory function on nutrition and gastrointestinal complaints in the elderly”	Denture retention affected nutritional status. Chewing problems, denture discomfort, or ill-fitting dentures increased incidence of food avoidance and digestive complaints of older adults.
Tooth loss		
Cross-sectional study (Singh et al., 2012) [62]	“Chewing disability in older adults attributable to tooth loss and other oral conditions”	In older adults, chewing ability was associated with loss of functional teeth (odds ratio: 4.2), dental pain (odds ratio: 4.88), and hypermobile teeth (odds ratio: 4.7).
Systematic review (Zelig et al., 2022) [64]	“Tooth loss and nutritional status in older adults: A systematic review and meta-analysis”	Malnutrition was associated with loss of functional teeth (odds ratio: 1.21) in older adults.
Randomized clinical trial (McKenna et al., 2012) [68]	“Impact of tooth replacement strategies on the nutritional status of partially-dentate elders”	Prosthodontic rehabilitation improved nutritional status of older adults.
Hyposalivation		
Cross-sectional study (Iwasaki et al., 2016) [66]	“Hyposalivation and dietary nutrient intake among community-based older Japanese”	Low intake of n-3 polyunsaturated fatty acid, potassium, vitamin D, vitamin E, vitamin B6, and folate increased risk of hyposalivation of older Japanese.

## Data Availability

Not applicable.
